# Synthesis of Levulinate Esters Using MgAl-Mixed Oxides Containing Transition Metals as Catalysts

**DOI:** 10.3390/molecules31101661

**Published:** 2026-05-14

**Authors:** Tanya Stoylkova, Tsveta Stanimirova, Kristina Metodieva, Christo D. Chanev

**Affiliations:** 1 Department of Mineralogy, Petrology and Economic Geology, Faculty of Geology and Geography, University of Sofia “St. Kliment Ohridski”, 15 Tsar Osvoboditel Blvd., 1504 Sofia, Bulgaria; stanimirova@gea.uni-sofia.bg (T.S.); k.metodieva@gea.uni-sofia.bg (K.M.); 2 Department of Organic Chemistry, Faculty of Chemistry and Pharmacy, University of Sofia “St. Kliment Ohridski”, 1 James Bourchier Blvd., 1164 Sofia, Bulgaria; hchanev@chem.uni-sofia.bg

**Keywords:** esterification, alkyl levulinates, isoamyl levulinate, cyclohexyl levulinate, n-butyl levulinate, mixed oxides, biomass

## Abstract

This study presents the production of isoamyl, n-butyl and cyclohexyl esters of levulinic acid with an excellent yield under solvent-free conditions. The catalysts used were MgAlO and M^2+^MgAlO-mixed oxides containing the transition metals (M^2+^ = Co^2+^, Ni^2+^, Zn^2+^), obtained from calcined layered double hydroxides (LDH). They are easily accessible, low-cost, and environmentally friendly and possess the requisite acid–base properties for esterification reactions. The effect of reaction time and the molar ratio of levulinic acid to the alcohols used on the esterification reaction was investigated. The catalysts were characterized by X-ray diffraction (XRD), XRF, SEM and temperature-programmed desorption of CO_2_ (TPD-CO_2_). Gas chromatography–mass spectroscopy (GC/MS) was used for the identification and quantification of the product mixtures. Mixed oxides containing transition metals exhibited significantly higher activity than MgAlO. Under the selected reaction conditions, the conversion of levulinic acid and the yield of isoamyl ester reached 100% at a reagent ratio of 1:1. As a by-product of esterification, only dicyclohexyl ether was found at a reactant ratio of 1:1.5.

## 1. Introduction

The global population is growing, which is leading to an increased demand for energy and raw materials by the chemical industry. The focus of recent chemical syntheses of major industrial interest has been on the use of renewable resources or waste. In this regard, lignocellulosic biomass has emerged as a sustainable alternative feedstock for energy and chemical production [1]. Lignocellulose, the most abundant type of biomass, primarily consists of cellulose, hemicellulose, and lignin. In addition, lignocellulosic biomass possesses a net-zero carbon footprint, balancing CO_2_ emissions through its fixation [2]. Levulinic acid (LA) is generally produced by acid-catalyzed hydrolysis of lignocellulosic biomass and can catalytically be transform to levulinate esters [3,4], γ-valerolactone [5], methyl ethyl ketone [6], 1,4-pentanediol and 5-nonanone (via pentanoic acid) as well as diphenolic acid as an intermediate for the synthesis of epoxy resins and poly-carbonates [7,8,9,10]. Among those, levulinate esters possess great commercial importance due to their potential applications as/in fuel-blending components [11], bio-lubricants [12], polymer precursor [13], environmentally friendly solvents [14,15], food-flavor agent [16], degreasing surface agent/stain removal [17], etc.

There are several methods for synthesizing levulinic esters, but esterification is the most direct approach. The reaction of LA with alcohols occurs at room temperature, but it is very slow and produces only a small amount of the final product [18]. Acceleration can be achieved by applying high temperatures (70–100 °C) or by using catalysts to quickly reach equilibrium conversion [19,20]. In this method, the alcohols act as both reagents and solvents, which are inherently polar in nature [21].

In earlier studies, levulinate esters are produced by reacting LA with several alcohols using mineral acids, such as HCl, H_2_SO_4_, and H_3_PO_4_, as catalysts. Although this process yields high amounts of the respective products (>95%) in a short amount of time, it is accompanied by non-recyclability, harsh reaction conditions, and operational problems [22,23,24]. Therefore, there has been a recent trend toward replacing corrosive mineral acids with more sustainable alternatives. Heterogeneous recyclable catalysts are widely used due to their advantages, which include catalytic activity, resistance, stability of the reaction environment, reusability, ease of application, and environmental friendliness. Various catalysts have been investigated as potential agents for achieving levulinic acid esterification, including zeolites [25,26], acid resin [27,28], silica-based catalysts [29,30,31], sulfated ZrO_2_ [18,32,33], and nanomaterials [34,35]. These catalysts have been used to produce short-chain esters, primarily methyl and ethyl levulinates.

There is limited literature regarding the synthesis of long-chain alkyl or cyclic levulinates using Kegging-type phosphovanadotungstic acid, lipase or lipase immobilized on SBA-15 or a sulfated silica nanocatalyst [36,37,38,39]. Interest in long-chain levulinic esters stems from their structural similarity to biodiesel. Their long carbon chains also provide higher carbon content and hydrophobicity, thereby improving the energy density of biodiesel.

Mixed oxides (Mg_x_AlO_y_) have been studied in different biomass-upgrading reactions due to their low cost and high activity. Layered double hydroxide (LDH)-derived mixed metal oxides are obtained by calcining an LDH precursor at various temperatures. These oxides possess high surface area, phase purity, and structural stability [40,41]. In general, mixed oxides form with three types of active sites: (i) weak Brønsted basic sites (OH^−^ groups on the surface), (ii) medium-strength Lewis sites (Mg^2+^-O^2−^ and Al^3+^-O^2−^ acid–base pairs), and (iii) strong Lewis basic sites (O^2−^ anions) [42]. Introducing optimally selected transition metals into the structure of mixed Mg-Al oxides can create different active sites, thereby increasing the catalyst’s flexibility [43,44]. Furthermore, LDH-derived mixed metal oxides containing various transition metal cations, such as Ni^2+^, Zn^2+^, and Co^2+^, exhibit the original acid–base and reduction–oxidation properties of the MgAl system, depending on the incorporated metal species [45]. Introducing transition cations into Mg_x_AlO_y_ structures can also decrease the total concentration of basic centers. The number of medium-strength acid–base pairs increase with the introduction of M^2+^ cations due to their higher electronegativity compared to Mg^2+^ cations [46]. Thus, mixed oxides can catalyze various acid–base reactions, including esterification, transesterification, and Claisen–Schmidt condensations [47,48]. To our knowledge, the literature contains practically no detailed information on mixed oxides containing transition metals as catalysts for the esterification of levulinic acid. Therefore, this study demonstrates the potential of mixed oxides as environmentally friendly, inexpensive, selective and efficient solid-phase catalysts for the esterification of levulinic acid with isoamyl alcohol (2-methylbutanol), n-butyl alcohol, and cyclohexanol. The studied mixed metal oxides containing the transition metals Ni^2+^, Co^2+^, and Zn^2+^ were prepared by synthesizing LDH precursors via the co-precipitation method. After calcination of the initial LDHs, the resulting oxides were used as catalysts. The transition metals were chosen according to their well-known ability to incorporate into the structure of Mg-Al mixed oxides, where they form active centers with medium acid–base properties.

## 2. Results and Discussion

### 2.1. Characterization of Catalysts

**Figure 1:**
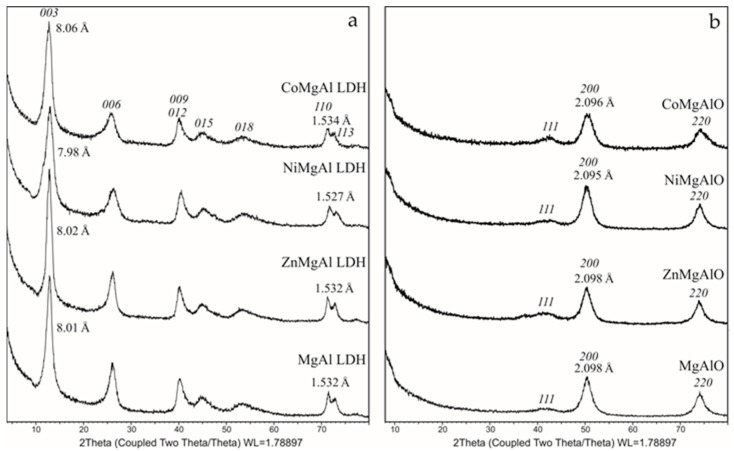
Powder XRD patterns of initial LDH (**a**) and their thermal decomposition products—mixed oxides (**b**).

The powder X-ray diffraction (XRD) results of the synthesized starting materials for the preparation of the mixed-oxide catalysts revealed that all the products were layered double hydroxides (Figure 1a). The values for the unit cell **a** parameter (a = 2d_110_) of the individual compositions denoted as MgAl-LDH, ZnMgAl-LDH, NiMgAl-LDH, and CoMgAl-LDH are 3.064 Å, 3.064 Å, 3.054 Å, and 3.068 Å, respectively. These values agree with the determined cationic chemical composition (Table 1). The values of the **c** parameter for all samples (c = 3d_003_) (Figure 1b) reflect the formation of the nitrate form of the obtained LDHs.

The predominant amount of Mg^2+^ (92–93%) as a divalent cation determines the formation of mixed oxides (denoted as MgAlO, ZnMgAlO, NiMgAlO, and CoMgAlO) with a periclase-type structure during the process of thermal decomposition of the starting LDHs (Figure 1b).

The crystallite sizes calculated using the Scherrer Equation (1) for the mixed oxides containing transition metals are similar (76 nm, 74 nm, and 77 nm for ZnMgAlO, NiMgAlO, and CoMgAlO, respectively), while for the MgAlO sample the crystallites are slightly larger (90 nm).

(1) $$D=\frac{K\lambda }{\beta \cos\Theta },$$

where *D* is crystallites size (nm), *K* is Scherer’s constant, *λ* is wavelength of the x-ray irradiation (1.78897), *β* is full width at half maximum in radians and *Θ* is peak position (in radians).

**Figure 2:**
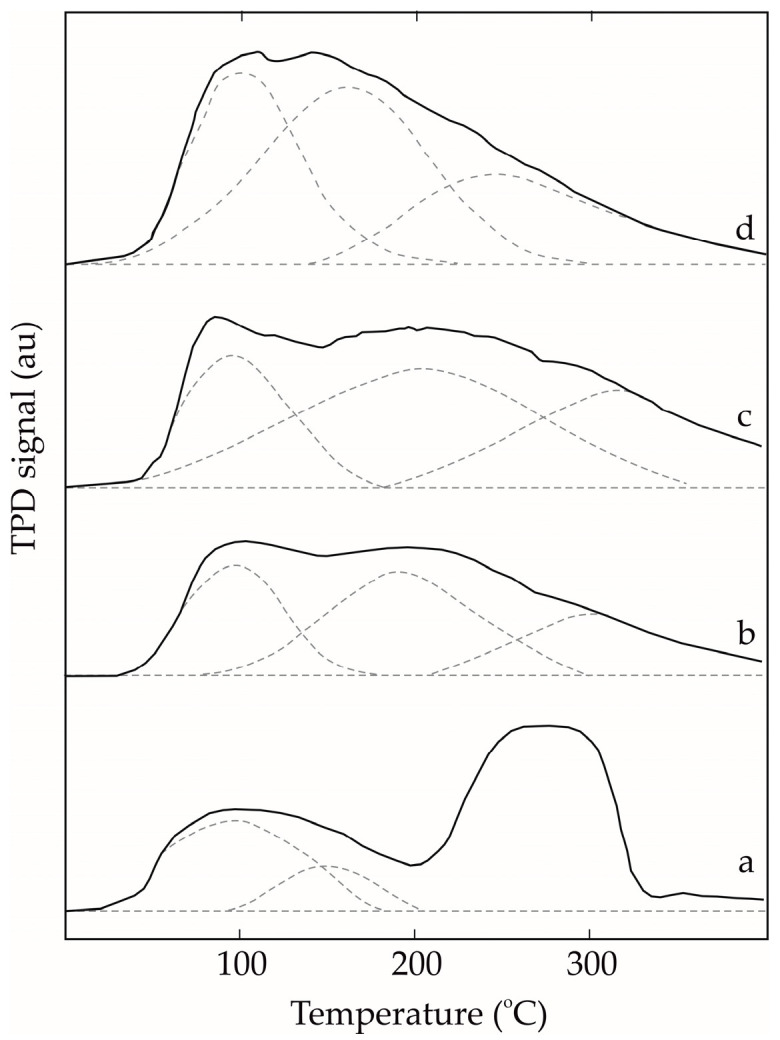
TPD—CO_2_ profiles of the mixed-oxide catalysts: (**a**) MgAlO, (**b**) CoMgAlO, (**c**) ZnMgAlO, (**d**) NiMgAlO.

The basic properties of the catalysts used were evaluated using CO_2_-TPD (Figure 2). The total concentration of basic centers (TB, in mmol/g) and the distribution of sites with different strengths are shown in Table 1. The resulting profiles of CoMgAlO, ZnMgAlO, and NiMgAlO are qualitatively similar. They can be deconvoluted into three desorption bands that reach maximum desorption rates at approximately 100 °C, 150–200 °C, and 250–300 °C. The MgAlO pattern is distinct from those of the other mixed oxides, exhibiting two clear peaks at approximately 100–150 °C and 300 °C. The amount of CO_2_ evolved on the MgAlO sample (125.2 mmol/g) was higher than on the M^2+^MgAlO samples (M^2+^ = Co, Zn, Ni). Based on our TPD results, we can describe the surface basicity of the mixed oxides as follows: “weak” basic sites for CO_2_ desorption at about 100 °C, “medium” basic sites for CO_2_ desorption between 150 °C and 200 °C, and “strong” basic sites above 250 °C [43]. Mixed oxides containing Lewis’s acid centers, such as CoMgAlO, ZnMgAlO and NiMgAlO, have predominantly weak and medium basic centers, with strong basic centers being present in much smaller quantities. By contrast, MgAlO, which contains the less electronegative Mg^2+^ cation compared to the Co^2+^, Zn^2+^ or Ni^2+^ cations, showed higher peaks corresponding mainly to strong-strength and weak sites (Table 1). In summary, introducing transition cations into M^2+^MgAlO structures modifies the acid–base properties of mixed oxides by decreasing the total concentration of strong basic centers and increasing the concentration of medium centers from 10% up to almost 50%. The weak centers show the smallest change in percentage.

### 2.2. Catalytic Activity

The esterification of levulinic acid with isoamyl alcohol, n-butyl alcohol and cyclohexanol was performed using MgAlO, CoMgAlO, NiMgAlO and ZnMgAlO-mixed oxides as catalysts. As is well known, mixed Mg/Al oxides with various Mg/Al ratios have been used for the esterification of benzoic acid with 2-ethylhexanol. The catalyst with a 4:1 Mg/Al ratio exhibited the highest activity, achieving a 66% conversion of benzoic acid to 2-ethylhexyl benzoate at 110 °C with a 10 wt. % catalyst loading, an acid-to-alcohol ratio of 1:5.6, and a 10 h reaction time [48]. It has been established that the presence of acid–base centers with appropriate strength significantly enhances acid conversion. The introduced transition metals have a higher Lewis acidity than magnesium ions and therefore participate in generating active acid–base centers of medium and weak strength. These metal ions can be coordinated directly to the carbonyl oxygen, increasing the positivity of the carbonyl carbon for subsequent nucleophilic attack [25]. The effectiveness of CoMgAlO, NiMgAlO and ZnMgAlO as catalysts for the esterification of levulinic acid was evaluated using a mixed oxide of MgAlO for comparison. To optimize process efficiency and avoid the progression of side reactions and by-product formation, mole ratios of 1:1 and 1:1.5 of LA to alcohol were used under identical experimental conditions. The products obtained were the corresponding levulinate esters. Traces of dicyclohexyl ether were only found at a molar ratio of LA/cyclohexanol of 1:1.5. The conversion of levulinic acid in the presence of the catalysts used is shown in Table 2.

As can be seen from the data (Table 2), MgAlO shows a significantly lower conversion rate compared to CoMgAlO, NiMgAlO and ZnMgAlO. A conversion of up to 100% is reached after a three-hour reaction time in the case of esterification of LA with isoamyl alcohol (1:1) over all M^2+^MgAlO samples and in the case of LA with n-butyl alcohol over CoMgAlO. Better results in terms of levulinic acid conversion were obtained at a 1:1 molar ratio of reactants, compared to a 1:1.5 ratio.

#### 2.2.1. Esterification of Levulinic Acid with Isoamyl Alcohol

**Figure 3:**
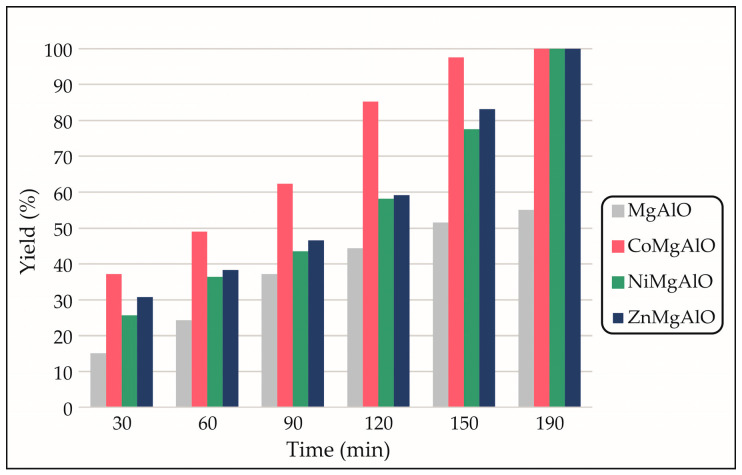
Yield of isoamyl levulinate as a function of time. Molar ratio 1:1, reaction temperature 120 °C.

**Figure 4:**
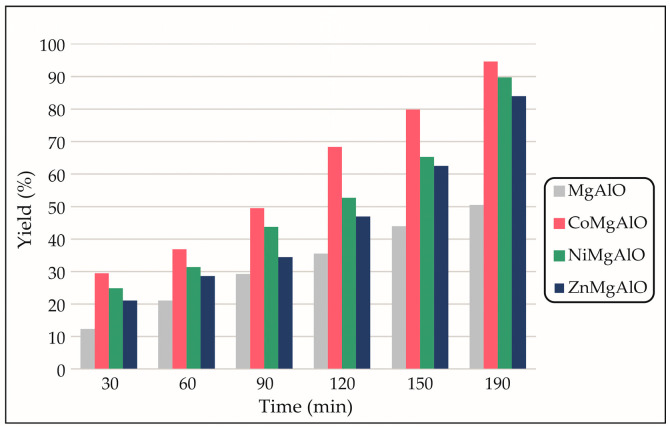
Yield of isoamyl levulinate as a function of time. Molar ratio 1:1.5, reaction temperature 120 °C.

The esterification of levulinic acid with isoamyl alcohol led to the formation of isoamyl levulinate, and no by-products were detected. Figure 3 and Figure 4 show the yields of the isoamyl ester obtained using the two molar ratios of 1:1 and 1:1.5, respectively.

During the reaction, CoMgAlO is the most active, followed by NiMgAlO and ZnMgAlO. However, these reach a product yield of 100% at the end of the third hour, with a 1:1 molar ratio of reagents. MgAlO, which is more strongly basic, shows significantly lower activity, with a product yield of around 50%.

As shown in Figure 4, the conversion of levulinic acid at a ratio of 1:1.5 is lower, reaching 94.2% with the most active catalyst (CoMgAlO).

#### 2.2.2. Esterification of Levulinic Acid with n-Butanol

**Figure 5:**
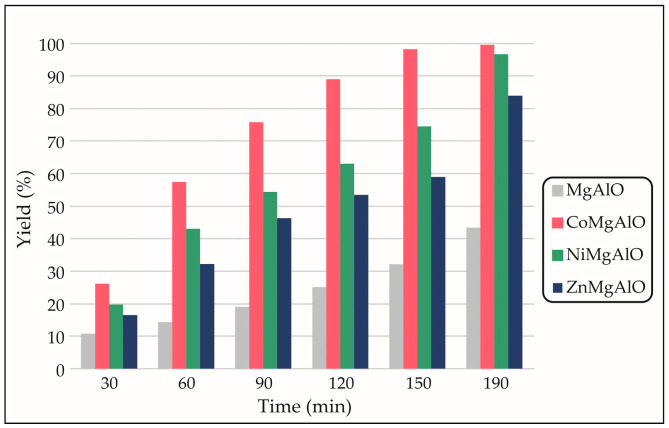
Yield of n-butyl levulinate as a function of time. Molar ratio 1:1, reaction temperature 120 °C.

**Figure 6:**
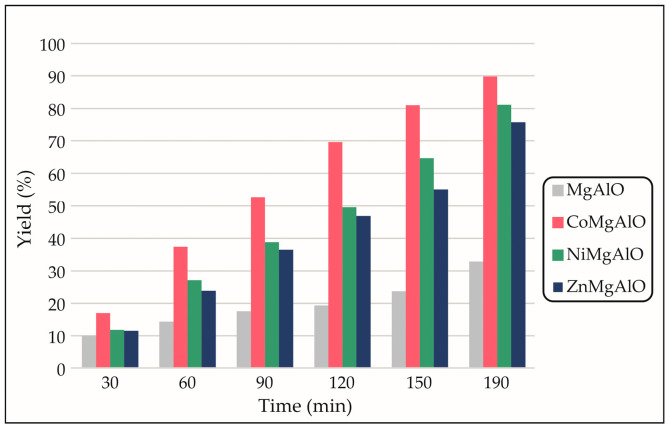
Yield of n-butyl levulinate as a function of time. Molar ratio 1:1.5, reaction temperature 120 °C.

The yields of n-butyl levulinate are shown in Figure 5 and Figure 6. In these cases, CoMgAlO also exhibits exceptional activity, achieving a yield of 100% (LA: alcohol = 1:1). NiMgAlO is slightly less active. ZnMgAlO shows a significant decrease in activity, with a yield of 84.3%.

At a ratio of 1:1.5, the yield of the product decreases for all catalysts used. Compared to the 1:1 ratio, the most significant decrease in product quantity was observed for NiMAlO, at 16.3%. Once again, the more basic MgAlO-mixed oxide exhibits significantly lower activity than oxides containing transition metals. The yield of n-butyl levulinate over MgAlO at a ratio of 1:1.5 is significantly lower, reaching only 32.6%.

#### 2.2.3. Esterification of Levulinic Acid with Cyclohexanol

**Figure 7:**
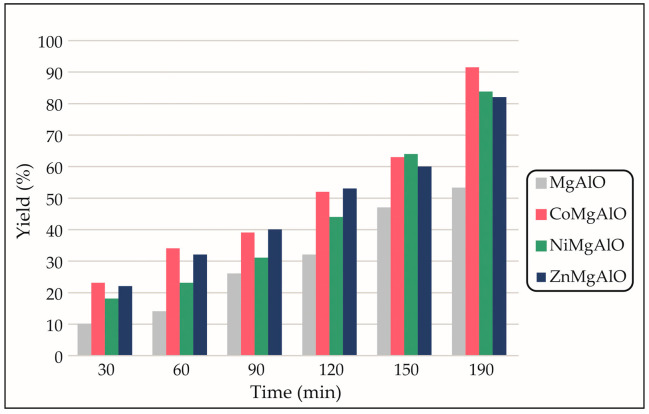
Yield of cyclohexyl levulinate as a function of time. Molar ratio 1:1, reaction temperature 120 °C.

When levulinic acid is esterified with cyclohexanol in a 1:1 molar ratio, only cyclohexyl levulinate is obtained (Figure 7).

**Figure 8:**
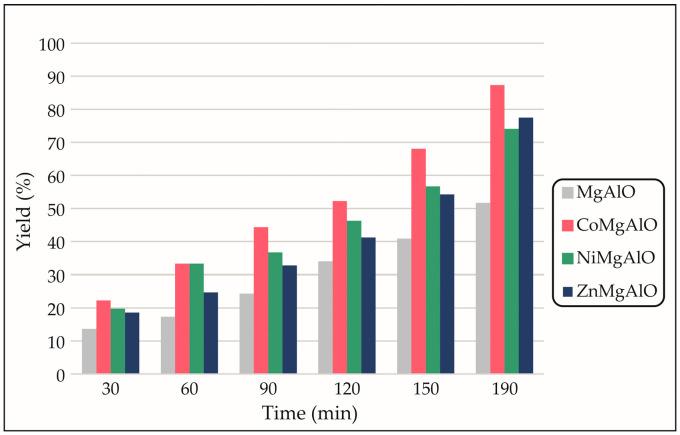
Yield of cyclohexyl levulinate as a function of time. Molar ratio 1:1.5, reaction temperature 120 °C.

The maximum yield was 91.6% when using CoMgAlO. At a molar ratio of 1:1.5, the product yield decreased to 71.1% and 63.1% in the presence of CoMgAlO and ZnMgAlO, respectively (Figure 8). Additionally, the by-product dicyclohexyl ether was produced, reaching a maximum amount of 1.9% at the end of the experiment over NiMgAlO.

The catalytic activity of the M^2+^MgAlO sample in esterification of LA with medium- and long-chain alcohols is compared to that of the other solid catalysts, such as SBA-15-APTS-GLU-Lip, heteropolyacid H_4_[PVW_11_O_40_], p-TSA and montmorilonite K10, and the results are presented in Table 3 [18,36,38,49]. The comparison demonstrates that the M^2+^MgAlO sample is more catalytically active than the other catalysts, such as the heteropolyacid H_4_[PVW_11_O_40_] and SBA-15-APTS-GLU-Lip in the synthesis of butyl, isoamyl, and especially cyclohexyl levulinates. The catalysts used offer several advantages, including an excellent conversion rate and the production of no by-products at a 1:1 ratio. The reactions are solvent-free, and the environmentally friendly catalysts can be easily and quickly synthesized from readily available raw materials.

The first amounts of by-product were detected from the 90th minute until the end of the experiments, in the presence of all catalysts. The levels were as follows: MgAlO: 0.3%, 0.4%, 0.8%, 1.3%; CoMgAlO: 0.1%, 0.5%, 0.6%, 1.0%; NiMgAlO: 0.2%, 0.4%, 0.9%, 1.9%; and ZnMgAlO: 0.4%, 0.5%, 0.9%, 1.2%. Ether was also observed as a by-product during the synthesis of cyclohexyl levulinate using p-TSA as a catalyst in an MW-assisted reaction at 200 °C with a LA:hexanol molar ratio of 1:6 [49].

**Scheme 1:**
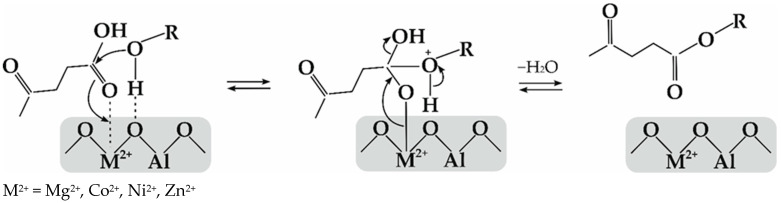
Plausible mechanism of the esterification reaction over mixed oxides containing transition metals.

The activity of the catalysts used for the esterification of levulinic acid can be explained by the following scheme, which is depicted in Scheme 1. The mixed oxides with containing transition metals are the more active with respect to the esterification product in compare to MgAlO catalyst. According to the TRD results, this is probably related to the decreasing of the concentration of strong basic centers and increasing the concentration of medium centers associated with M^2+^–O^2−^ (M^2+^ = Co^2+^, Ni^2+^, Zn^2+^) active pairs. Among the mixed oxides containing transition metals, CoMgAlO shows the highest activity, followed by NiMgAlO and finally ZnMgAlO.

M^2+^MgAlO with improved acid–base pairs of the appropriate strength coordinate levulinic acid’s carbonyl oxygen, thus increasing electrophilicity of the carboxylic carbon. At the same time, bridge oxygens form a stable hydrogen bond by abstraction of a proton from the hydroxyl group of alcohol. This spatial arrangement favors a nucleophilic attack by the alcohol, leading to the formation of a tetra-coordinated, sp^3^-hybridized intermediate. The next step is dehydration and the formation of the carbonyl group of the target ester.

The results obtained are consistent with the acidity of the alcohols used, as this most likely affects the yield of the levulinic esters produced. From this point of view, the esterification of LA with cyclohexanol (secondary alcohol) leads to lower conversion and yield of cyclohexyl ester compared to n-butyl and isoamyl alcohols. Spatial constraints related to access to the active sites may also arise when esterification proceeds with neighboring sites on the catalytic surface participating.

**Figure 9:**
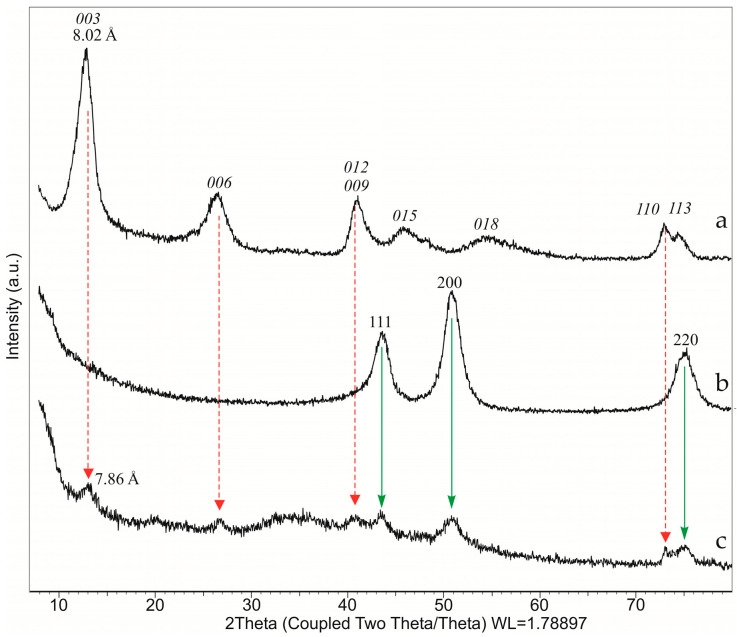
Powder XRD patterns of initial NiMgAl-LDH (**a**); mixed oxide NiMgAlO before (**b**); and after (**c**) catalytic reaction.

It is known that water is one of the products of esterification. The high conversion of levulinic acid and the high yield of the corresponding esters are probably because the catalyst absorbs the separated water, which leads to the conversion of the mixed oxides back into layered double hydroxides. Clear evidence of this restoration of the layered double hydroxide structure was obtained by XRD of the used catalysts (Figure 9). In powder XRD pattern of spent catalysts, besides mixed oxide, X-ray reflections of newly formed LDH are also registered. The observed smaller parameter c (c = 3d_003_ = 23.58 Å) suggests formation of a OH form of the LDH (Figure 9), which is consistent with the absence of nitrate ion in the system during the catalytic reaction.

Removing the water released during esterification prevents reverse hydrolysis, thereby shifting the reaction equilibrium toward the products. Although excess alcohol should shift the equilibrium toward the product, our results showed a decrease in conversion. Similar results are described in the literature we cite [18]. The reduced conversion may be due to dilution or water retention in the reaction mixture, which shifts the equilibrium back. At a molar ratio of 1:1.5, conversion is lower, most likely because the reaction medium is diluted. At the same time, the presence of small amounts of dicyclohexyl ether suggests that etherification is occurring as a side reaction. The appearance of the ether no earlier than 90 min after the start of the experiments indicates that its formation may involve Brønsted acid sites formed on the catalytic surface due to the water released during esterification. This provides sufficient hydroxyl groups on the surface of the recrystallized LDH. Therefore, the side reaction is more likely to occur on recrystallized LDH than on the initial mixed oxides. Therefore, in this case, the ether can be obtained in the traditional way, first by binding a proton from the Brønsted center to form an oxonium ion. This is then followed by an interaction with a new cyclohexanol molecule to produce the final dicyclohexyl ether. No clear correlation was observed between the strength of the acid–base pairs and transition metals of the catalysts used and the amount of dicyclohexyl ether formed.

## 3. Materials and Methods

### 3.1. Synthesis of the Catalysts

The LDH precursors of the catalysts used were prepared by a co-precipitation method at room temperature [8]. The M^2+^MgAl-LDH were synthesized by the following procedure: Aqueous solutions as Mg(NO_3_)_2_•6H_2_O and Al(NO_3_)_3_•9H_2_O ((M^2+^ + Mg)/Al = 3 at 1 M) were added dropwise at a rate of 600 rpm into a beaker containing transition metal nitrate solution such as Co(NO_3_)_2_•6H_2_O, Ni(NO_3_)_2_•6H_2_O and Zn(NO_3_)_2_•6H_2_O. The pH was maintained at 10 ± 0.2 with an appropriate volume of 1 M NaOH solution. The M^2+^/(M^2+^ + Mg + Al) atomic ratio was around 0.05, and the transition metal content was 10% in terms of cations. The resulting precipitates were aged in their mother liquor at 90 °C in an air atmosphere for 24 h, cooled to room temperature, filtered and washed repeatedly with distilled water to remove residual nitrates and sodium and to achieve a pH of 7. The solids were then dried in an oven at 90 °C for 18 h. The corresponding mixed oxides (M^2+^MgAlO) were then obtained by calcination at 500 °C for 2 h in air. Finally, all the mixed oxides were finely ground in an agate mortar and stored in a desiccator (Kartell 550, KartellLABWARE, Noviglio, Italy).

### 3.2. Characterization of the Catalysts

Powder X-ray diffraction measurements were performed using a BRUKER instrument (Billerica, MA, USA) with filtered Co-Kα radiation to study the structures of the catalysts. The instrument was operated with 0.02° (2θ) steps within the 4–80° (2θ) angular range, with a counting time of 1.5 s per step. Specialized Difraction, EVA software, version 5.2.0.5, was used to determine the qualitative phase composition. Morphology was investigated using a JEOL JSM-6010PLUS/LA scanning electron microscope (SEM) (Akishima, Japan) fitted with an energy-dispersive spectrometer (EDS). The metal content (Mg, Al, Zn, Co, and Ni) of the catalyst samples was determined by XRF using an Omnian 3SW Epsilon 3XLE instrument (Malvern Panalytical, Malvern, UK). The measurements were conducted on glass disks of lithium borates. The results are summarized in Table 1.

The number of basic sites in the mixed oxides used was determined by identifying the peaks in the CO_2_-TPD curve that corresponded to the desorption of CO_2_ from these sites. The total amount of CO_2_ desorbed was then calculated by integrating the area under these peaks. This value was then used to calculate the number of basic sites on the mixed oxides. Each desorbed CO_2_ molecule corresponds to one basic site on the material. Temperature-programmed desorption (TPD) of 10% CO_2_ in argon was performed using an ABB AO2040 infrared gas analyzer (Frießnitz, Germany). The TPD was performed under conditions where diffusion limitations and re-adsorption could be neglected. All TPD experiments were conducted using a 0.05 g sample with an average particle size of 250–500 µm. The carrier gas flow velocity was 25 mL/min, with a linear temperature rise rate of 25 °C/min. Prior to the TPD experiments, the samples were pretreated by increasing the temperature to 350 °C in an argon atmosphere. This temperature was then maintained in the gas environment for one hour. The catalyst was then cooled to room temperature. After pre-treatment of the samples, the following steps were performed: (i) adsorption of CO_2_ at room temperature for 30 min; (ii) temperature-programmed desorption (TPD) from room temperature to 350 °C; (iii) slow cooling of the catalyst to room temperature; (iv) cleaning of the catalyst surface with argon for about 30 min; (v) adsorption of CO_2_ at 100 °C for 30 min; (vi) slow cooling of the catalyst to room temperature; (vii) cleaning of the catalyst surface with argon for about 30 min; (viii) adsorption of CO_2_ at 100 °C for 30 min; and (ix) TPD from 100 °C to 350 °C.

### 3.3. Reaction Procedure

Each mixed oxide catalyst was activated at 350 °C for 1 h before the reaction. The esterification of levulinic acid (0.05 mol) with isoamyl alcohol (0.05 mol) or n-butyl alcohol or cyclohexanol and powder catalyst (0.5 g) was studied under solvent-free conditions. The molar ratio of LA to alcohol was 1:1 or 1:1.5. The weight ratio of the catalyst to the alcohols was approximately 1:20 (1:1 molar ratio of reagents) and 1:25, 1:23, and 1:27 (1:1.5 molar ratio) for isoamyl alcohol, n-butyl alcohol, and cyclohexanol, respectively. The reactions were carried out in a round-bottom flask fitted with a water-cooled reflux condenser. The resulting mixture was rapidly heated to 120 °C under vigorous stirring (600 rpm) in an oil bath equipped with an automatic temperature control system. The reaction time for all experiments was three hours, with samples being taken from each experiment every 30 min.

Gas chromatography–mass spectroscopy (GC/MS) was used to identify and quantify the reaction products. The GC/MS analyses were performed on an HP 6890 chromatograph with an HP 5973 mass selective detector (Agilent, Santa Clara, CA, USA). The column used was HP-5/MS, 30 m × 0.250 mm × 0.25 µm. The mass balances, calculated after a reaction time of 1 h, were always higher than 95%.

## 4. Conclusions

This study demonstrates that mixed oxides of M^2+^MgAlO produced by calcining layered double hydroxides containing transition metals (M^2+^ = Co^2+^, Ni^2+^, Zn^2+^) can be used as effective and selective catalysts in esterification reactions. We produced isoamyl, n-butyl and cyclohexyl levulinates, which are commonly used as diesel fuel additives. Better results in terms of conversion and product yield were obtained at a 1:1 reagent ratio in the presence of CoMgAlO, followed by NiMgAlO and ZnMgAlO. The excellent yield of up to 100% after three hours is due to the availability of acid–base pairs of the appropriate strength and the complete esterification reaction. This reaction occurs when the released water combines with the mixed oxides, which removes the reaction mixture. The mixed oxides then recrystallize into layered double hydroxides. The only by-product obtained in very small quantities was dicyclohexyl ether. This was produced at a reactant molar ratio of 1:1.5 after 90 min from the start of the reaction.

## Figures and Tables

**Table 1:** The chemical composition and characteristics of the basic sites of mixed oxides.

Catalyst	M^2+^/(M^2+^ + Mg + Al) Atomic Ratio		(M^2+^ + Mg)/Al Atomic Ratio		TB * mmol/g	Distribution of Basic Sites (mmol/g)		
	Theor.	Exp.	Theor.	Exp.		Weak (%)	Medium (%)	Strong (%)
MgAlO	-	-	3	3.24	125.2	41.2 (32.9)	12.6 (10.1)	71.4 (57.0)
CoMgAlO	0.05	0.06	3	3.44	81.6	24.1 (29.5)	40.2 (49.3)	17.3 (21.2)
ZnMgAlO	0.05	0.05	3	3.25	104.3	30.2 (29.0)	49.0 (47.0)	25.1 (24.0)
NiMgAlO	0.05	0.06	3	3.14	96.4	32.2 (33.4)	44.1 (45.8)	20.1 (20.8)

* TB—total basicity; M^2+^ = Co^2+^, Zn^2+^, Ni^2+^; M^2+^ = Mg^2+^ for MgAlO.

**Table 2:** Conversion of LA (%), molar ratio LA/alcohol—1:1 and 1:1.5, time 3 h, reaction temperature 120 °C.

Catalysts	Conversion (%)					
	LA/Isoamyl Alcohol		LA/n-Butyl Alcohol		LA/Cyclohexanol	
	1:1	1:1.5	1:1	1:1.5	1:1	1:1.5
MgAlO	55.1	50.3	43.5	32.6	53.3	43.3
CoMgAlO	100	94.2	100	89.4	91.6	72.1
NiMgAlO	100	89.4	97.1	80.8	83.9	62.2
ZnMgAlO	100	87.6	84.3	75.4	82.1	64.3

**Table 3:** Conversion of LA over different solid catalysts in the esterification of LA with medium and long-chain alcohols.

Catalyst	Conversion (%)	Product	Reaction Conditions			
			Temperature (°C)	Reagent Ratio	Reaction Time (h)	Amount of Catalyst
SBA-15-APTS-GLU-Lip [38]	85	1-butyl levulinate	50	LA/1-butanol = 1:2	2.5	5 wt. %
	94	isoamyl levulinate		LA/isoamyl alcohol = 1:2		
	90	1-hexyl levulinate		LA/1-hexanol = 1:2		
H_4_[PVW_11_O_40_] [36]	94	1-butyl levulinate	85	LA/1-butanol = 1:8;	1.5 or 4	LA/catalyst = 0.01 and 0.03
	99	isoamyl levulinate		LA/cyclohexanol = 1:8		
	21	cyclohexyl levulinate		LA/isoamyl alcohol = 1:8		
p-TSA [49]	98	1-hexyl levulinate	200	LA/1-hexanol = 1:6	0.05 + MW	5 wt. %
montmorillonite K10 [18]	96.6	1-butyl levulinate	170	LA/1-butanol = 1:25	2.25	50 mg; N_2_ pressure 0.6 MPa
this paper M^2+^MgAlO	100	isoamyl levulinate	120	LA/alcohol = 1:1	3	5 wt. %
	100	n-butyl levulinate				
	92	cyclohexyl levulinate				

## Data Availability

Data is contained within the article.
